# A Peculiar Deformity

**Published:** 1878-07

**Authors:** H. A. Kiker

**Affiliations:** Tallapoosa, Ga.


### A PECULIAR DEFORMITY.

By H. A. KIKER, M.D., Tallapoosa, Ga.

Deeming it the duty of every member of the profession to report all cases that would be of interest to its members, I send you the report of a case which is a freak of nature entirely new to me, as it will perhaps be to the profession generally.

While on a visit to this place last August I was called on to remove what I pronounced a supernumerary nose on an infant three weeks old. It was a small pear-shaped organ, situated on the right nasal bone near the inner canthus of the eye, with the base downward. It consisted of skin, soft cartilage and mucous membrane, and was about the size of a natural nose of an infant of that age. It was connected with the right nasal cavity by two openings, these running into one, near the lower end of the organ, that would only admit a small probe to be passed through it. The air passed through the organ while breathing—the right canal of the natural nose not being as well developed as the left, owing, as I think, to a portion of the air passing through this extra nose. It was not called into use equally with the left side, or, in other words, there were two air passages on that side to do the work of one. I removed this supernumerary nose by making an incision from the internal and inferior surface upwards and outwards, making a semi-circular flap of skin from superior and external side of the organ to cover the wound; had considerable hemorrhage for a few seconds from a small artery that supplied the organ, but it was easily controlled by pressure. The flap was secured by one suture. The operation was performed without anæsthetics, by the infant being held on the lap of an assistant. I did not visit the case after the operation, but learned that the wound healed by the first intention, and that the child is doing well up to the present time.

